# miR-223 increases gallbladder cancer cell sensitivity to docetaxel by downregulating STMN1

**DOI:** 10.18632/oncotarget.11634

**Published:** 2016-08-26

**Authors:** Wei Lu, Yunping Hu, Qiang Ma, Linzhu Zhou, Lin Jiang, Zhizhen Li, Shuai Zhao, Yuzhen Xu, Weibin Shi, Sheng Li, Yingbin Liu

**Affiliations:** ^1^ Department of General Surgery, Xinhua Hospital, Affiliated to Shanghai Jiao Tong University, School of Medicine, Shanghai, China; ^2^ Institute of Biliary Tract Diseases Research, Shanghai Jiao Tong University School of Medicine, Shanghai, China; ^3^ Institute of Chemistry, Shanghai Jiao Tong University, Shanghai, China; ^4^ Department of Gastrointestinal Surgery, Xu Zhou Center Hospital, Affiliated to Medical College of Southeast University, Jiangsu, China; ^5^ Department of Biochemistry, Dalian Medical University, Liaoning, China

**Keywords:** miR-223, gallbladder cancer, malignancy, STMN1

## Abstract

**Background:**

MicroRNAs (miRs) are involved in cancer carcinogenesis, and certain regulatory miRs could provide promising therapeutic methods for refractory malignancies, such as gallbladder cancer (GBC). miR-223 was found to play a pivotal role in enhancing chemotherapeutic effects, therefore evoking interest in the role of miR-223 in GBC.

**Results:**

miR-223 was decreased in GBC tissues and cell lines, and ectopic miR- 223 expression exhibited multiple anti-tumorigenic effects in GBC cells, including decreased proliferation, migration and invasion *in vitro*. However, treatment with a miR-223 inhibitor increased cell viability. We determined that STMN1 was negatively correlated with and regulated by miR-223 in GBC. miR-223 increased GBC sensitivity to docetaxel *in vitro* and *in vivo*, and the induced sensitivity to docetaxel was suppressed by the restoration of STMN1 expression.

**Methods:**

We examined miR-223 expression in GBC tissue and GBC cell lines using qRT-PCR. The effects of modulated miR-223 expression in GBC cells were assayed using Cell Counting Kit-8 (CCK8), flow cytometry, and wound-healing and invasion assays. Susceptibility to docetaxel was evaluated in miR-223/STMN1-modulated GBC cells and xenograft tumor models. The protein expression of relevant genes was examined by Western blotting.

**Conclusions:**

These findings indicated that miR-223 might serve as an onco-suppressor that enhances susceptibility to docetaxel by downregulating STMN1 in GBC, highlighting its promising therapeutic value.

## INTRODUCTION

Gallbladder cancer (GBC) is rare; however, it is the most common malignancy of the biliary tract [1]. Cancers within this tract are difficult to treat because of their highly lethal nature [2, 3]. Patients with GBC have a reported overall 5-year survival of less than 5% and a mean survival of only 6 months [4], and their initial clinic presentation is often at an advanced stage of the disease [5, 6]. Moreover, studies reporting the benefit of neoadjuvant therapy for patients with advanced GBC failed to provide sufficient power to show significant improvements [7]. Controlling the progression of advanced GBC remains unfavorable, which contributes to the current poor prognosis of patients with GBC.

MicroRNAs (miRs) are noncoding 17 to 25 nucleotide RNAs that post-transcriptionally regulate gene expression [8]. These RNAs are believed to be expressed in a tissue-specific manner and play important roles in cell proliferation, apoptosis, and differentiation during mammalian development [9] as well as oncogenesis and tumor metastasis [10, 11]. Aberrant expression of certain miRs have been shown to promote cancer initiation and progression by modulating their target genes [12], thus designating these miRs as cancer-related miRs [13–15]. It was hypothesized that manipulating miR expression would change their biological behaviors and contribute to the treatment of corresponding malignancies [16, 17]. Some preclinical studies have demonstrated that modulating miR expression levels could increase chemotherapy efficacy [18, 19] and have highlighted potential applications to improve the treatment of certain chemo-resistant malignancies [20].

miR-223 was first reported to be involved in the regulation of human granulocyte proliferation and function [21, 22]. Some studies have demonstrated that miR-223 plays a complicated role in leukemia [23, 24]. miR-223 was shown to be significantly downregulated in progressive chronic lymphocytic leukemia [25–27]. Recent studies further investigated the downregulation of miR- 223 in chronic lymphocyte leukemia [28], nasopharyngeal carcinoma [29], hepatocellular carcinoma [30], gastric cancer [31], malignant pleural mesothelioma [32] and prostate cancer [33] via different mechanisms. Moreover, miR-223 was demonstrated to rescue the cellular response to anticancer drugs by modulating ABCB1 (ATP-Binding Cassette Sub-Family B Member 1) in hepatocellular carcinoma [34] and targeting PARP1 (poly(ADP-ribose) polymerase 1) in esophageal adenocarcinoma [35].

The present study was performed to investigate the role of targeted miR-223 treatments on the invasion and metastasis of GBC cells and to explore the potential chemo-sensitizing effect of miR-223. Our results indicate that miR-223 may be involved in GBC development, whereby GBC cells overexpressing miR-223 exhibit increased sensitivity to chemotherapy agents. These results provide valuable information for potential clinical applications.

## RESULTS

### Aberrant miR-223 expression in GBC tissue samples and correlation with STMN1 upregulation

**Figure 1:**
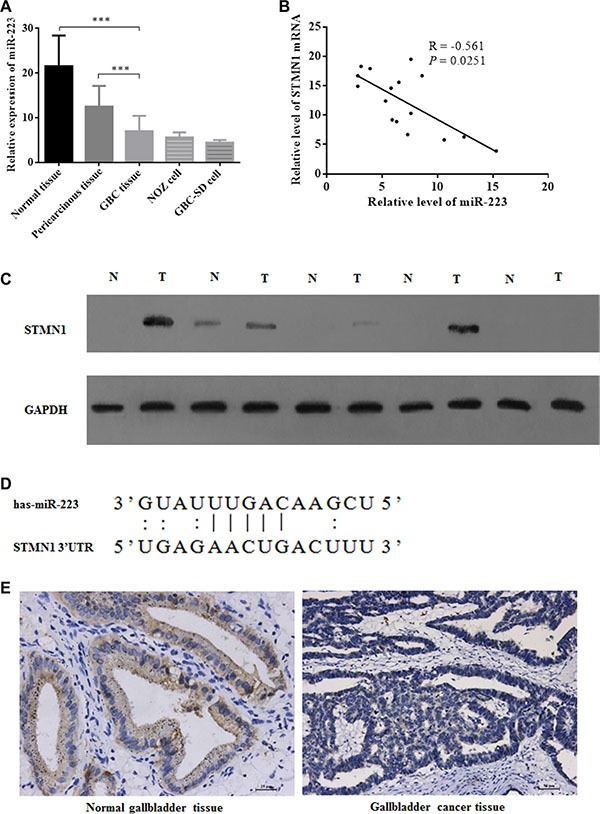
Aberrant miR-223 expression in gallbladder cancer tissue samples and correlations with STMN1 upregulation (**A**) qRT-PCR detection of miR-223 expression in 5 normal gallbladder tissues, 16 gallbladder cancer tissues and their matched pericarcinous gallbladder peripheral tissues, 2 gallbladder cancer cell lines. miR-223 expression was significantly higher in normal gallbladder tissues (*P* = 0.0002) and peripheral tissues from GBC patients (*P* = 0.0003) but was downregulated in GBC tissue. The data are presented as the mean ± SD from three independent experiments. (**B**) The inverse correlation between miR-223 and STMN1 mRNA expression in gallbladder cancer tissue samples (*n* = 16) by linear regression analysis. (**C**) A Western blot examining the protein expression level in the tissue samples of 5 gallbladder cancer samples and their peripheral tissues. (**D**) Sequence alignment of miR-223 with the 3′ UTR of the STMN1 gene. (**E**) miR-223 expression in normal (left) and cancerous (right) gallbladder tissues examined by *in situ* hybridization.

To determine the mRNA and protein expression levels of miR-223 in GBC, quantitative real-time RT-PCR (qRT-PCR) and Western blot assays were performed, respectively. The expression of miR-223 and STMN1 mRNA in the surgically resected tissue samples from 5 cholecystolithiasis patients and 16 GBC patients, and 2 GBC cell lines was investigated. miR-223 was highly expressed in non-cancerous gallbladder tissues and in pericarcinous gallbladder tissues from GBC patients but was downregulated in GBC tissues and cell lines (Figure 1A). The expression of STMN1 mRNA and miR- 223 in 16 GBC cancer tissues was also examined. STMN1 mRNA expression was significantly negatively correlated with miR-223 expression in GBC patients (Figure 1B). Therefore, we further examined STMN1 protein levels by Western blotting and qRT-PCR in 5 pairs of GBC tissues with their pericarcinous gallbladder tissues and observed that the expression levels of STMN1 protein were elevated in GBC tissues (Figure 1C and Supplementary Figure S2). These results suggested that miR-223 was downregulated in GBC and correlated with elevated STMN1 expression. We then performed a bioinformatic analysis of potential miR-223 target genes using the online miRBase sequence database provided by the University of Manchester [36]. STMN1 was predicted as one of the target genes with a PicTar score of 3.12; the putative target sites for miR-223 in the 3′ UTR of the STMN1 mRNA are shown in Figure 1D. miR-223 was downregulated in GBC tissue compared with normal gallbladder tissue as determined using *in situ* hybridization (Figure 1E).

### miR-223 mimics and inhibitors efficiently elevate and decrease miR-223 levels, respectively, in GBC cells to modulate STMN1 expression

**Figure 2:**
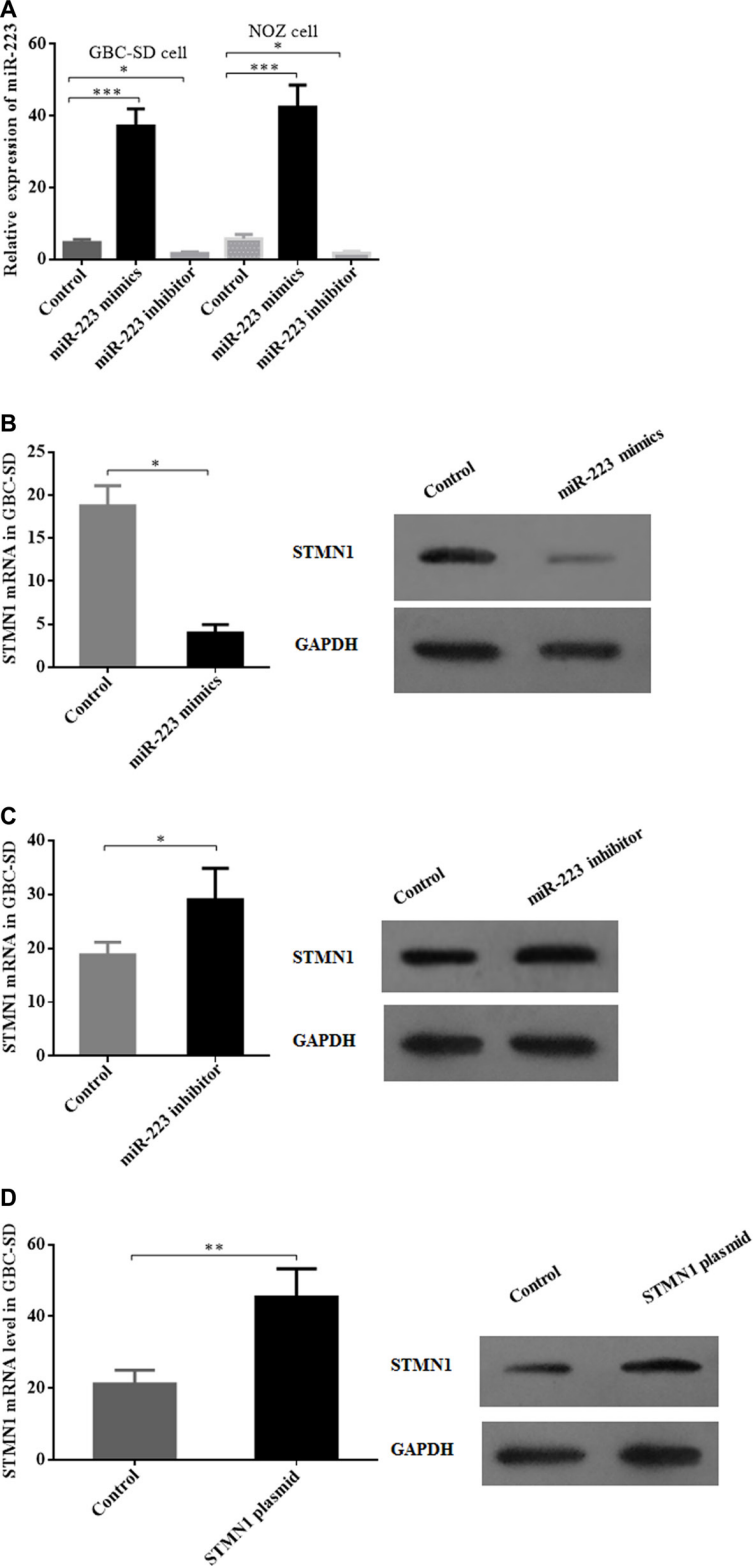
Modulation of miR-223 and STMN1 expression in gallbladder cancer cells by miR-223 mimics, a miR-223 inhibitor and a STMN1 overexpression plasmid (**A**) miR-223 levels in GBC-SD and NOZ cell lines were significantly elevated upon transfection of a miR-223 mimics vector and decreased by a miR-223 inhibitor. (**B**) Both the STMN1 mRNA and protein expression levels were decreased after the transfection of miR-223 mimics in GBC-SD cells. (**C**) Both STMN1 mRNA and protein expression levels were increased after transfection of a miR-223 inhibitor in GBC-SD cells. (**D**) STMN1 expression was significantly increased after transfection of a STMN1 expression plasmid. The expression of miR-223 and STMN1 mRNA was measured by qRT-PCR and the expression of STMN1 protein by Western blotting.

To observe the effect of modulating the miR-233 levels and STMN1 expression in GBC cells, we used miR-223 mimics, a miR-223 inhibitor and an STMN1 expression plasmid to transfect GBC-SD and NOZ cells. In the GBC-SD and NOZ cell lines, qRT-PCR analysis showed that miR-223 expression was efficiently elevated or decreased 24 h after transfection of miR-223 mimics or miR-223 inhibitor, respectively, compared with the control group (Figure 2A). The STMN1 mRNA and protein levels were simultaneously modulated with miR-223 mimics, miR-223 inhibitor and the STMN1 expression plasmid in GBC cells (Figure 2B–2D and Supplementary Figure S1).

### Ectopic miR-223 suppresses GBC cell proliferation, whereas a miR-223 inhibitor promotes GBC proliferation

**Figure 3:**
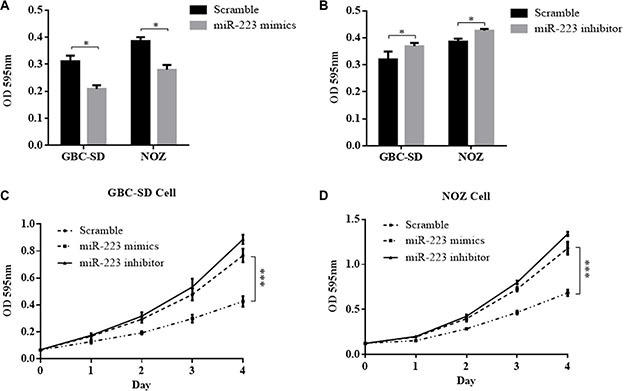
The effect of miR-223 mimics and inhibitor on GBC cell proliferation (**A**) Overexpression of miR-223 suppressed cell growth in GBC-SD and NOZ cells. (**B**) Inhibition of miR-223 stimulated cell growth in GBC-SD and NOZ cells. (**C**) and (**D**) The effect of overexpression and inhibition of miR-223 on the cell growth curve of GBC-SD and NOZ cells. At 24 h after transfection of the indicated vector, GBC-SD and NOZ cells were seeded into 96-well cell culture plates. The proliferative effects were evaluated by CCK8 assay 48 h later, as shown in (A) and (B). Cell viability was measured every 24 h using a CCK8 assay as shown in (C) and (D). The data are presented as the mean ± SD from three independent experiments.

To investigate the biological function of miR-223 in GBC development and progression, we examined cell proliferation using the Cell Counting Kit-8 (CCK8) assay. At 2 days after the introduction of exogenous miR-223, GBC-SD and NOZ cell proliferation was significantly lower in cells treated with miR-223 mimics compared with that of the scramble controls by 32.9% and 27.5%, respectively, (*P* < 0.05, Figure 3A). By contrast, GBC-SD and NOZ cell proliferation was significantly higher upon treatment with the miR-223 inhibitor compared with that of the scramble controls by 15.2% and 10.4%, respectively (*P* < 0.05, Figure 3B). The growth curve of the GBC cells after transfection with either exogenous miR-223 mimics or inhibitor was evaluated in GBC-SD and NOZ cells. GBC cell growth was significantly faster when transfected with miR-223 inhibitor but was significantly slowed in the presence of the miR-223 inhibitor compared with the cells transfected with control vector (*P* < 0.001 for both, Figure 3C and 3D).

### miR-223 overexpression inhibits GBC cell migration and invasion

**Figure 4:**
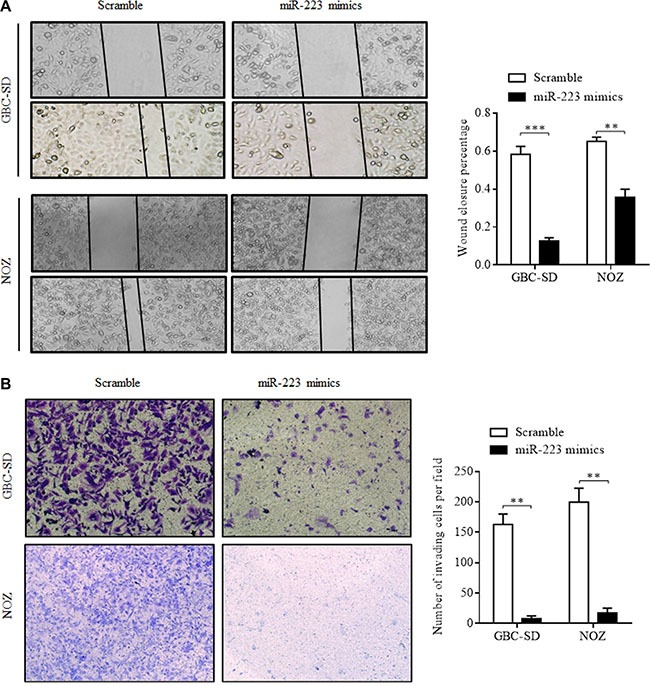
Overexpression of miR-223 inhibits GBC cell migration and invasion (**A**) The migratory ability of GBC-SD and NOZ cells transfected with miR-223 mimics was assessed using a wound-healing migration assay. Representative phase-contrast photomicrographs and wound-closure rates are shown at 0 and 24 h after wound formation. (**B**) The invasive ability of GBC-SD and NOZ cells transfected with miR-223 mimics was assessed using a transwell invasion assay. Following a 24-h incubation, invasive cells that passed through the Matrigel chambers were fixed and stained; these cells and the cell migration rates are shown. The error bars represent the mean ± SD of triplicate experiments (**P < 0.01; ***P < 0.001).

Wound-healing and invasion assays were performed in GBC cells to evaluate the effect of miR-223 on cell migration and invasion. The results showed that transfection of miR-223 mimics reduced the wound-healing distance at 24 h compared with the scramble control (78.3% of GBC-SD cells and 45.2% of NOZ cells, *P* < 0.001, Figure 4A). In the cell invasion transwell assay, the number of cells that migrated through the Matrigel-coated membrane into the lower chamber (invasion assay) was significantly lower among cells transfected with miR-223 mimics than in scramble-transfected cells. The transfection of miR-223 mimics inhibited 95.1% of GBC cell (*P* < 0.001) and 91.3% NOZ cell (*P* < 0.001) migration compared with the scramble control (Figure 4B). These data suggest that exogenous miR-223 might inhibit GBC cell metastasis.

### GBC-SD and NOZ cell resistance to docetaxel was sensitized by transfection of miR-223 mimics

**Figure 5:**
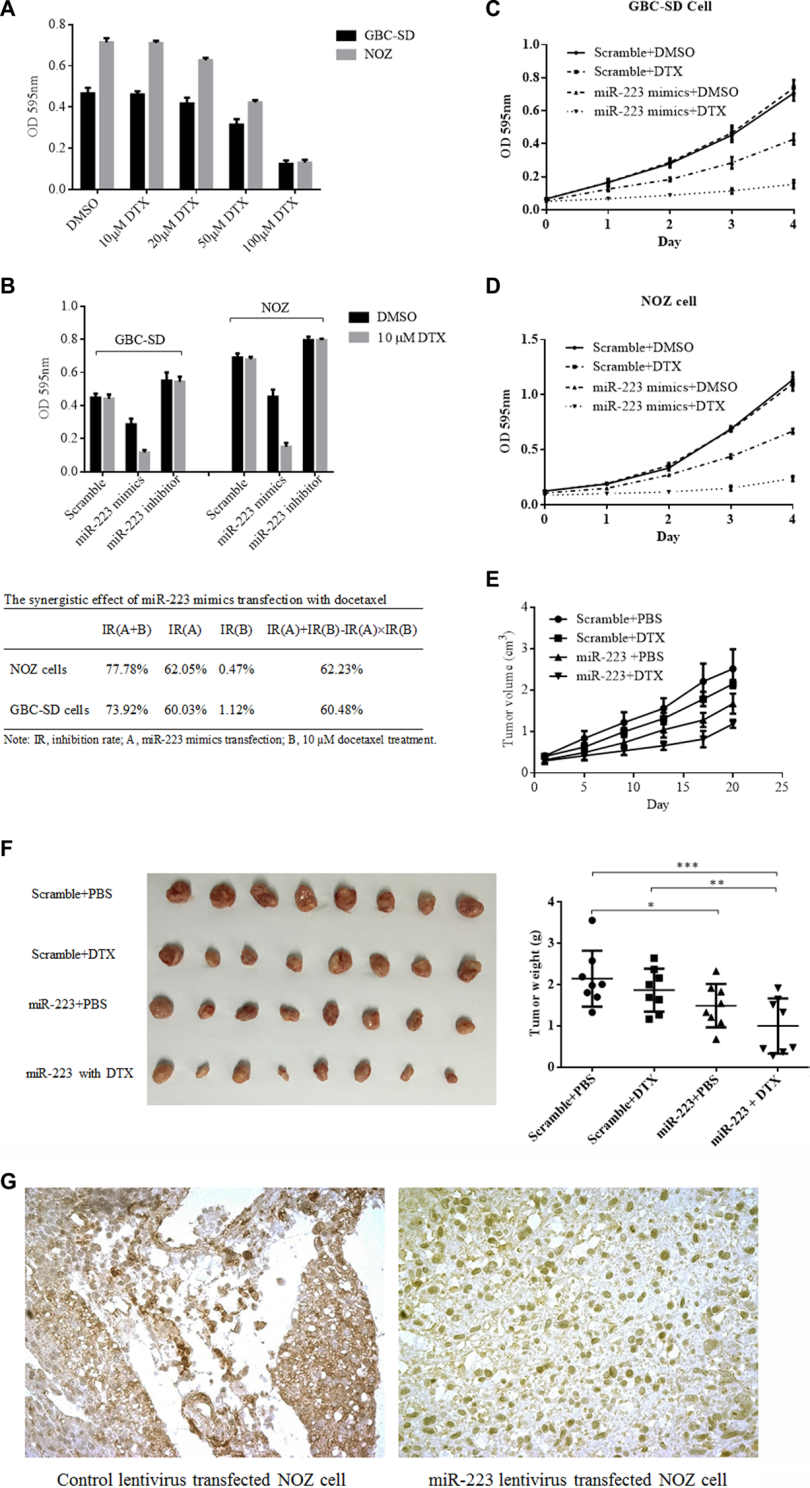
GBC-SD and NOZ cells were resistant to docetaxel and sensitized by ectopic expression of miR-223 (**A**) GBC-SD and NOZ cells were resisted to 100 μM docetaxel (DTX). Either GBC-SD or NOZ cells were seeded into 96-well cell culture plates for 24 h, then treated with DMSO or docetaxel; the cytostatic effects were evaluated by CCK8 assay. (**B**) miR-223 mimics enhanced the cytotoxicity of docetaxel in GBC-SD cells and NOZ cells. After 24 h of transfection, GBC-SD or NOZ cells were seeded in 96-well cell culture plates and treated with either DMSO or 10 μM docetaxel for an additional 48 h followed by a CCK8 assay. (**C**) and (**D**) A low concentration of docetaxel was lethal to GBC-SD cells and NOZ cells overexpressing miR-223. At 24 h after transfection, GBC-SD and NOZ cells were seeded into 96-well cell culture plates with either DMSO or 10 μM docetaxel, and cell viability was measured every 24 h using a CCK8 assay. (**E**) (**F**) and (**G**) Overexpression of miR-223 inhibited tumor growth and induced sensitivity to docetaxel in the NOZ xenograft model. There were four groups of mice: scramble control with either PBS or DTX and miR-223 mimics with either PBS or DTX. The tumor growth curves are shown in (E). The mice were sacrificed, and the tumors were harvested and weighed 21 days after the initial injection (*P < 0.05; **P < 0.01; ***P < 0.001) as shown in (F). The harvested tumor tissues were examined for STMN1 expression by immunohistochemistry. (G) NOZ cells lentivirally transduced with scramble control (left side) or exogenous miR-223 (right side).

GBC is a refractory malignancy that is resistant to most chemotherapy agents. The literature has failed to provide strong evidence that patients with advanced GBC could benefit from neoadjuvant chemotherapy [7]. Upon discovering that miR-223 might downregulate STMN1 and inhibit GBC proliferation, STMN1 activity was reported to be correlated with certain chemotherapy agents [37], suggesting that the downregulation of STMN1 could sensitize malignant cells to docetaxel [38]. Therefore, we hypothesized that miR-223 could downregulate STMN1 expression and subsequently increase the sensitivity of GBC cells to chemotherapy agents that target microtubules, such as docetaxel. We first investigated the sensitivity of GBC-SD and NOZ cells to docetaxel using the CCK8 proliferation assay. The results showed that both cell lines were resistant to docetaxel below a concentration of 50 μM, with an IC_50_ value of 82.43 μM for GBC- SD cells (95% CI, 75.66 to 89.81) and an IC_50_ value of 68.5 for NOZ cells (95% CI, 63.90 to 73.43) as shown in Figure 5A. Next, we investigated the sensitivity of GBC cells transfected with either miR-223 mimics or an inhibitor using the CCK8 proliferation assay. We observed that the GBC cells were sensitized to 10 μM docetaxel upon transfection of miR-223 mimics. We also compared the cytostatic effect of 10 μM docetaxel in conjunction with miR-223 mimic transfection to docetaxel treatment alone, and the results showed that GBC cells transfected with miR-223 mimics had a significantly higher cytostatic effect upon treatment with 10 μM docetaxel compared with that of the GBC cells transfected with scramble. The inhibition rate was increased by 72.80% in the GBC-SD cells (*P* < 0.001) and 77.31% in the NOZ cells (*P* < 0.001). Moreover, the inhibition rate was significantly higher in cells transfected with miR-223 mimics treated with 10 μM docetaxel than in cells that were only transfected (73.92% vs. 36.37%, respectively, for the GBC-SD cells (*P* < 0.001) and 77.78% vs. 34.34%, respectively, for the NOZ cells (*P* < 0.001)). No significant differences were detected in the cells transfected with the miR-223 inhibitor. The possibility of synergy was evaluated using the formula IR(A+B)>IR(A)+IR(B)-IR(A) × IR(B), where IR is the inhibition rate. Figure 5B shows synergistic effects were observed in the GBC-SD and NOZ cells. We further investigated the growth curve of GBC-SD and NOZ cells after transfection with miR- 223 mimics and in the presence or absence of docetaxel. GBC cell growth was significantly suppressed when treated with 10 μM docetaxel and transfected with miR-223 mimics compared with that of the other groups for both cell lines (*P* < 0.001, Figure 5C and 5D). These data suggest that exogenous miR-223 could increase GBC cell sensitivity to cancer chemotherapy. Next, we observed the chemosensitizing effect of the miR- 223 mimics vector *in vivo* using a NOZ xenograft model. The tumor volume growth was significantly slower in animals grafted with NOZ cells transduced with lentivirus expressing miR- 223 expression compared with that of the other animals (*P* < 0.001, Figure 5E). The measured tumors were significantly smaller in the miR- 223-expressing NOZ- cell group than those in the scramble vector group (Figure 5F). STMN1 expression in the tumor was examined by immunohistochemistry and showed weaker staining in miR-223-expressing tumors than in scramble control tumors (Figure 5G).

### The chemosensitizing effects of miR-223 mimics were neutralized by restoring STMN1 expression using a transfected STMN1 overexpression plasmid

**Figure 6:**
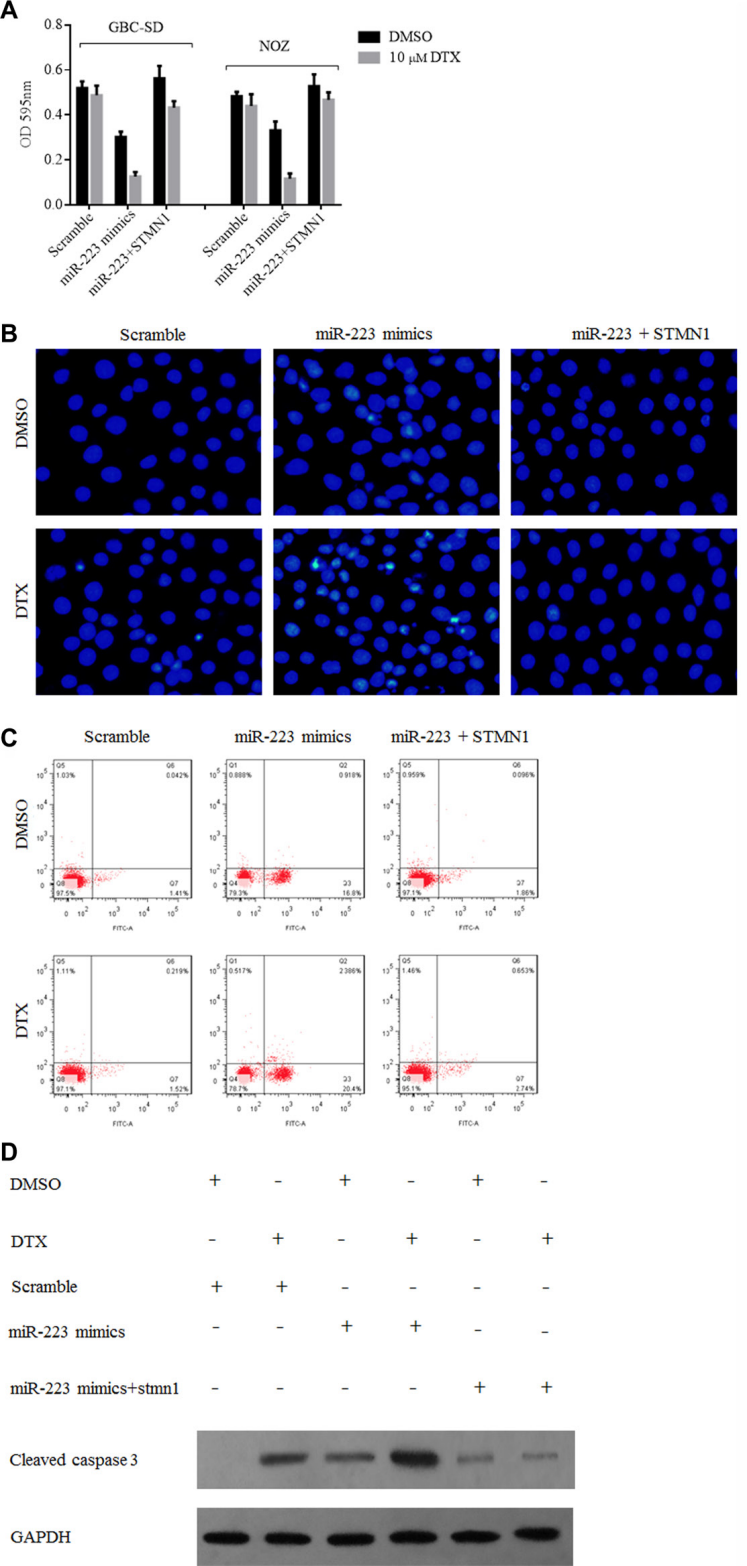
The cytotoxic effect of docetaxel to gallbladder cancer cells overexpressing miR-223 was suppressed by STMN1 restoration (**A**) Transfection of an STMN1-expressing plasmid reversed the growth inhibition of docetaxel in gallbladder cancer cells overexpressing miR-223. GBC-SD and NOZ cells were transfected with indicated vector and seeded into 96-well cell culture plates for 24 h. The cells were then treated with either DMSO or 10 μM docetaxel and cultured for another 48 h before they were subjected to a CCK8 assay. The data are presented as the mean ± SD from three independent experiments. (**B**) and (**C**) Overexpression of miR-223 increased apoptosis of GBC-SD cells. However, this effect was suppressed by transfection of a STMN1 expression vector. GBC-SD cells were transfected with the indicated vector for 24 h, after which, the cells were seeded into 6-well cell culture plates. The following day, cells were treated with either DMSO or 10 μM docetaxel for 24 h. The extent of apoptosis was measured using an Annexin V-FITC Apoptosis Detection kit by flow cytometry as shown in panel (B) and DAPI-stained the nuclei of GBC-SD cells as shown in panel (C). (**D**) Overexpression of miR-223 increased docetaxel-induced caspase 3 cleavage, whereas co-transfection of the STMN1-expressing plasmid suppressed this effect in GBC-SD cells.

The above results demonstrated that ectopic miR-223 expression exerts a chemosensitizing effect by downregulating STMN1. Therefore, we used an STMN1 vector to restore its expression in GBC cells. The response of the treated GBC-SD and NOZ cells to docetaxel was evaluated using the CCK8 assay, with cell apoptosis quantified by DAPI and Annexin V/PI staining and flow cytometry and by evaluating the expression level of cleaved caspase 3 by Western blotting. Cellular resistance to docetaxel was restored after transfection of the STMN1 expression plasmid (Figure 6A). The number of apoptotic cells was increased in groups treated with docetaxel and expressing ectopic miR-223 compared with those expressing the scramble control, but cells co-transfected with STMN1 vector showed no significant change in the levels of apoptosis (Figure 6B and 6C). We also detected the expression of cleaved caspase 3 to evaluate the apoptotic activity of GBC-SD cells ectopically expressing miR-223 treated with docetaxel. Cleaved caspase 3 levels were increased in docetaxel-treated cells transfected with miR-223 but not in scramble-transfected cells; furthermore, STMN1 transfection suppressed this effect (Figure 6D).

## DISCUSSION

GBC is one of the intractable malignancies of the digestive system. Radical surgical resection is the sole promising treatment for GBC available to patients in the early stage of this disease. However, due to embryology origination and anatomy, GBC is difficult to diagnose in the early stages. Moreover, GBC barely responds to available anticancer regimens or radiotherapy due to its biological nature. miRs are small non-coding RNA molecules that function as negative regulators of mRNA and have been reported to exert important regulatory effects in carcinogenesis [39–44].

Accumulating evidence has supported important roles for miRs as either tumor suppressors or oncogenes [15]. The recent development of miR-based therapeutics has provided a new strategy in cancer treatment [45]. We observed that miR-223 was involved in diverse malignancies and induced a prominent reversal in chemotherapy resistance, which has triggered an interest in investigating the role of miR-223 in GBC.

To date, the consensus is that miR-223 expression is aberrant in multiple cancer types; however, the expression and function of miR-223 in tumors remains controversial. There are reports identifying miR-223 overexpression in gastric cancer [46], colorectal cancer [47] and esophageal squamous cell carcinoma [48], but with unknown mechanisms. These reports indicated the complicated role of miR-223 in carcinogenesis. Microtubules are dynamic α/GAPDH heterodimers that play key roles in cell division, morphology, motility, and intracellular transport [49]. STMN1 is a microtubule-regulatory protein that modulates microtubule dynamics by preventing tubulin polymerization and promoting the destabilization and disassembly of microtubules during interphase and late mitosis during the cell cycle progression; this process is regulated by changes in the phosphorylation status of STMN1 [50]. STMN1 also plays a role in a variety of other biological processes such as cell proliferation, mobility, metastasis, differentiation, and resistance to antimicrotubule therapy [51]. One previous study confirmed that STMN1 is a target of miR-223 and that downregulation of miR-223 contributes to chemoresistance in various cultured tumor cells [52]. Thus, we hypothesized that the ectopic expression of miR-223 could induce the chemosensitivity of GBC cells.

In the present study, we revealed that miR-223 is downregulated in human GBC and demonstrated that exogenous miR-223 expression inhibits GBC cell proliferation, induces GBC cell apoptosis, and suppresses GBC cell migration and invasion. Moreover, exogenous miR-223 sensitized GBC cells to chemotherapy reagents. miR-223 has multiple target genes, including STMN1, that function as microtubule modulators. Therefore, we further investigated and observed that STMN1 expression was modulated by transfection of either miR-223 mimics or an inhibitor in a GBC cell line. These results suggest that ectopic miR-223 expression may induce an anticancer effect by downregulating STMN1 expression in GBC cells. These data demonstrate for the first time that miR-223 functions as a tumor suppressor in GBC and that the miR-223/STMN1 pathway in GBC carcinogenesis is worthy of further investigation.

In summary, our study indicated that the introduction of ectopic miR-223 in GBC cells inhibited proliferation and reduced invasiveness and metastasis, thus enhancing the sensitivity of GBC to chemotherapy *in vitro* and *in vivo* and suggesting the possible application of miR- 223 as a therapeutic target for GBC. Further studies are required to fully understand the detailed mechanisms of miR-223 in GBC carcinogenesis and as a potential therapeutic approach.

## MATERIALS AND METHODS

### Reagents

The CCK8 assay was purchased from Dojindo Laboratories (Kumamoto, Japan). Puromycin, docetaxel (DTX), anti-STMN1 antibody and anti-GAPDH antibody were purchased from Sigma. Anti-Digoxin IgG monoclonal antibody was obtained from Invitrogen. Fetal bovine serum, DMEM and William's medium E cell culture medium were purchased from Gibco. The RIPA cell lysate buffer, bicinchoninic acid (BCA) assay and enhanced chemiluminescent (ECL) detection reagent were purchase from Cell Signaling, Thermo Scientific and Pierce, respectively. The oligonucleotides encoding the hsa-miR-223 mimics (miR-223), mimics control (miR-control), hsa-miR-223 inhibitor (anti-miR-223) and inhibitor control (anti-miR-control), as well as the STMN1 expression plasmid, were obtained from ZoonBio (Nanjin, China). The hsa-miR-223 overexpression lentivirus and digoxin-labeled miR-223 probe were purchased from Exiqon (Vedbaek, Denmark). An anti-caspase 3 antibody was purchased from Abcam. An apoptosis assay kit was purchased from Biotium Inc. Lipofectamine 2000 was obtained from Invitrogen. TRIzol, OPTI-MEM, M-MLV Reverse Transcriptase, pre-miR-223 and antisense nucleotides of miR-223 were purchased from Life Technologies. The qPCR primers were purchased from BBI Life Science Corporation, the TaqMan human MicroRNA Assay kits from Qiagen and SYBR^®^ Green PCR Master Mix from Applied Biosystems. The DAPI stain was purchased from Beyotime Biotechnology (Nantong, China), and Triton™ X-100 was obtained from Takara Bio (Dalian, China).

### GBC tissue samples and cell lines

Fresh samples of GBC tissues stored in liquid nitrogen and formalin-fixed as well as paraffin-embedded cancerous gallbladder tissue samples with a validated pathology diagnosis were obtained from the tissue sample library of the Department of General Surgery at Xinhua Hospital Affiliated to Shanghai Jiaotong University School of Medicine (Shanghai, China). Written consent was obtained after approval by the local Ethics Committee. None of the enrolled patients underwent either preoperative chemotherapy or radiotherapy.

The human GBC cell lines GBC-SD and NOZ were purchased from the Cell Bank of the Chinese Academy of Sciences (Shanghai, China). The GBC-SD cells were cultured in DMEM and the NOZ cells in William's medium E. The media for both cell lines were supplemented with 10% fetal bovine serum (FBS) and 1% penicillin-streptomycin (10,000 U/ml penicillin and 10 mg/ml streptomycin). The cell lines were incubated at 37°C in a humidified atmosphere with 5% CO_2_.

### Tissue immunohistochemistry for STMN1 and *in situ* hybridization for miRNA

Immunohistochemistry was performed to investigate STMN1 expression in the GBC tissues harvested from a xenograft mice model as previously described [53]. Briefly, the tissues were formalin-fixed, paraffin-embedded and cut into tissue sections. The tissue sections were then dehydrated with ethanol, washed three times with phosphate-buffered saline (PBS) and boiled for 8 min in a pressure cooker for antigen retrieval. Endogenous peroxidase activity was blocked by incubating in 3% hydrogen peroxide for 10 min at 26°C. The sections were further blocked with 3% normal goat serum for 10 min. After the serum was discarded, the sections were incubated overnight with primary rabbit anti-human STMN1 antibody in a humidified chamber at 4°C. The following day, the sections were incubated with secondary antibody-coated polymer peroxidase complexes (Abcam, Cambridge, UK) for 30 min at room temperature. After three 3-min washes with PBS, the sections were developed using diaminobenzidine (Abcam), and the slides were counterstained with hematoxylin for long-term storage. Negative controls were treated identically but without the primary antibody treatment.

The paraffin-embedded tissue section tissue sections were dehydrated with ethanol, washed 3 times with PBS and boiled for 15 min in a sodium citrate solution. Then, digested with 5 μg/ml proteinase K at 37°C for 5 min and washed 3 times with PBS for 3 min followed by incubation in 0.1 mol/L glycine PBS for 10 min and 0.25% acetic anhydride in 0.1 mol/L triethanolamine (pH 8.0) for 10 min. The sections were then pre-hybridized with 0.2× SSC and 50% formamide for 60 min at 37°C. Hybridization was performed with the probe in the hybridization solution for 18 h. Finally, the sections were washed using SSC and detected by immunohistochemistry with an anti-Digoxin IgG monoclonal antibody and horseradish peroxidase-conjugated secondary antibodies to detect miR-223 expression.

### Western blotting

After treatment, the cells were collected and lysed in RIPA buffer. After centrifugation at 14,000 × g for 30 min, the protein concentration of the harvested supernatant was determined using the BCA assay. The protein lysates (20 μg/lane) were separated by 10% SDS-polyacrylamide gels and then blotted onto polyvinylidene difluoride (PVDF) membranes. The membranes were blocked and then probed with the primary antibodies against either STMN1 or GAPDH. The membranes were then incubated with horseradish peroxidase-conjugated secondary antibodies. The immunocomplex was visualized using an enhanced chemiluminescent (ECL) detection reagent.

### RNA isolation and qRT-PCR

Total RNA was isolated from tissue samples and cell lines using TRIzol reagent. cDNA was synthesized from 2 μg of total RNA using random primers and M-MLV Reverse Transcriptase. The expression levels of the miRNAs and STMN1 mRNA were evaluated by qRT-PCR, with the U6 small nuclear RNA and GAPDH, respectively used for normalization. The following primers were used for the detection of miR-223, STMN1 and GAPDH expression according to the human STMN1 and GAPDH cDNA sequences in GenBank:

miR-223_F: 5′- GCGTGTATTTGACAAGCTGAG TT -3′;

miR-223_R: 5′- GTGTCAGTTTGTCAAATACCC CA -3′;

STMN1_F: 5′- GCCTGTCGCTTGTCTTCT -3′;

STMN1_R: 5′- TCATGGGACTTGCGTCTT -3′;

GAPDH_F: 5′- CAACAGCCTCAAGATCATCAGC -3′;

GAPDH_R: 5′-TTCTAGACGGCAGGTCAGGTC -3′;

U6_F 5′-CTCGCTTCGGCAGCACA-3′′ and

U6_R 5′-AACGCTTCACGAATTTGCGT-3′.

PCRs of each sample were conducted in triplicate. The relative expression level of the target gene was calculated using 2^−ΔCT^ (ΔCT = C_T_^STMN1^ - C_T_^GAPDH^) and normalized to the relative expression detected in the corresponding control tissue or cells. miR-223 expression was defined as upregulated when the relative expression ratio was > 1 and downregulated when the relative expression ratio was < 1.

### Cell biology experimental procedures

In these studies, the following cell biology experiments were performed: cell viability assay; transient transfection; flow cytometric cell cycle analysis; Annexin V/PI staining assay; wound-healing migration assay; transwell migration assay; and invasion assay. Generally, these experiments were performed as previously described [54].

### DAPI staining cell apoptosis test

Treated NOZ cells (1 × 10^5^ cells/well) were seeded in 24-well plates with round coverslips and exposed to docetaxel for 24 h. Following washing with PBS, the cells were fixed with buffered paraformaldehyde and incubated with 0.1% Triton™ X-100 at room temperature for 10 min. The cells were treated with DNase-Free RNase (50 mg/ml) for 2 h at 37°C and then stained with 5 μM DAPI for 5 min at room temperature. Cell apoptosis was assessed under a fluorescence microscope. The cells with a condensed nucleus were defined as apoptotic cells.

### Animal experiments

To investigate the effects of the indicated genes in tumor growth, NOZ cells were transfected with either the miR-223 overexpression lentivirus or scramble control lentivirus and selected with puromycin. Approximately 36 h after transfection, 5 × 10^6^ cells were suspended in serum-free medium and injected s.c. into nude mice (*n* = 8/group). At 10 days after the injection (when the subcutaneous tumor reached approximately 0.5 cm in diameter), either 100 μg of docetaxel (approximately 5 mg/kg) or the same volume of PBS was injected i.v. every week for 3 weeks. The tumor volume was measured every 3 days. The mice were sacrificed 21 days later, and the tumors were weighed. The animal experiments were conducted in strict accordance with the experimental animal guidelines and protocols, which were reviewed and approved by the Institutional Animal Care and Use Committee at Shanghai Jiao Tong University.

### Statistical analyses

Statistical analysis was performed using a GraphPad Prism 5 software package (GraphPad Software, San Diego, CA, USA). The results are presented as the mean ± standard deviation (SD). Differences between subgroups were tested using Student's *t*-test. Parameter correlation was tested using nonparametric Spearman correlation analysis and line fitting by linear regression. A *P*-value of less than 0.05 (denoted by *) was considered significant (**P < 0.01, ***P < 0.001).

## SUPPLEMENTARY MATERIALS FIGURES


