# Supplementary material for: miR-223 increases gallbladder cancer cell sensitivity to docetaxel by downregulating STMN1
Source: Oncotarget. 2016 Aug 26;7(38):62364–76. doi: 10.18632/oncotarget.11634 (PMC5308733; doi:10.18632/oncotarget.11634)
Supplement: Supplementary file 1 [file oncotarget-07-62364-s001.pdf]

miR-223 increases gallbladder cancer cell sensitivity to docetaxel by downregulating STMN1

Supplementary Materials

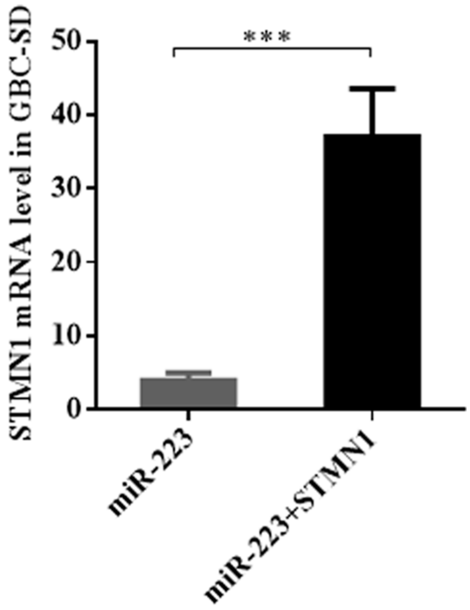

Supplementary Figure S1: Comparison of STMN1 expression levels in gallbladder cancer cells ectopically expressing miR-223 with or without STMN1 transfection.

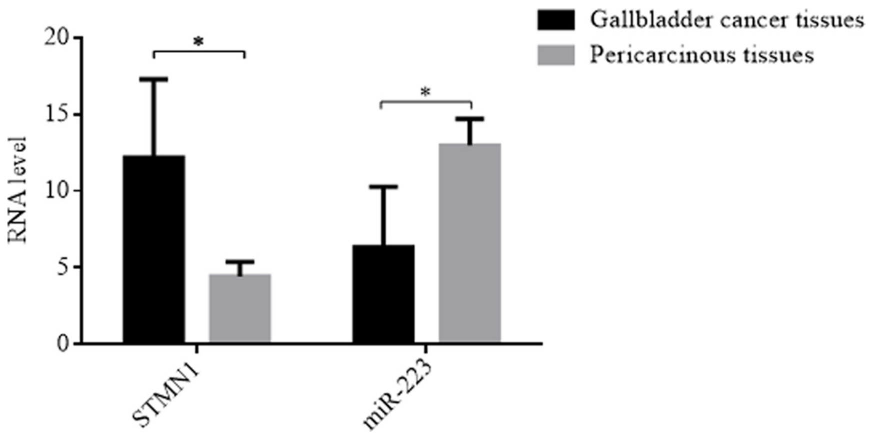

Supplementary Figure S2: We further examined the STMN1 and miR-223 RNA levels in 5 pairs of GBC tissues with their pericarcinous tissues. STMN1 mRNA was upregulated, whereas miR-223 levels were downregulated in GBC tissues compared with their pericarcinous tissues. We have added these results in the supplementary data section.

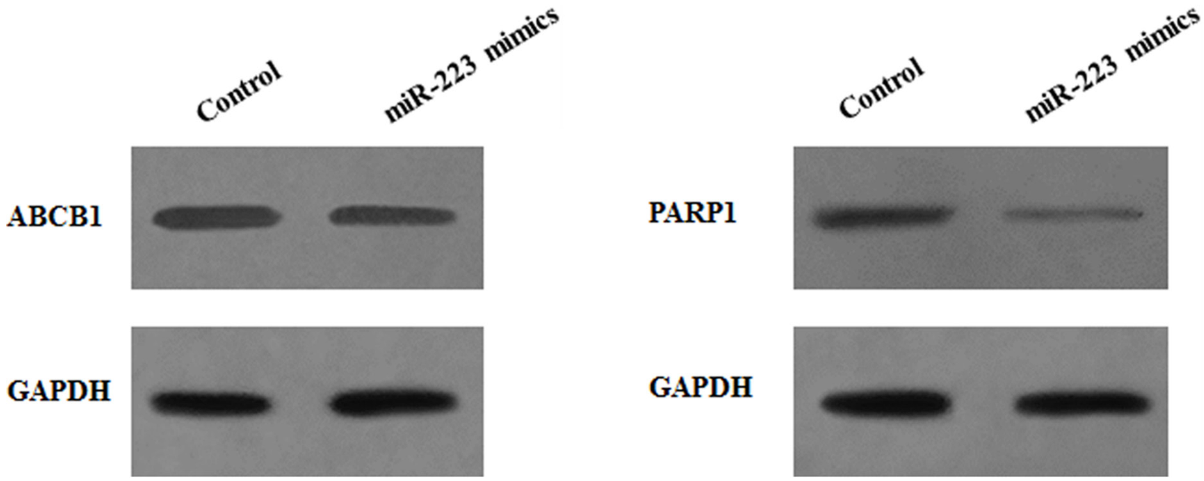

Supplementary Figure S3: Comparison of ABCB1 and PARP1 protein expression levels in NOZ gallbladder cancer cells expressing either scramble control or ectopic miR-23 by Western blotting.
